# Income loss among the self-employed: implications for individual wellbeing and pandemic policy measures

**DOI:** 10.1007/s11150-021-09583-6

**Published:** 2021-09-13

**Authors:** Stefan Schneck

**Affiliations:** 1 Institut für Mittelstandsforschung (IfM) Bonn, Maximilianstrasse 20, 53111 Bonn, Germany; 2 GLO, Leimkugelstr. 6, 45141 Essen, Germany

**Keywords:** L26, Entrepreneurial households, Income, Income cuts, Self-employment

## Abstract

Due to the pandemic-induced economic crisis, self-employed individuals are currently suffering considerable income losses. The self-employed and the members in their households usually form an economic unit. As a consequence, the income cuts not only affect the self-employed themselves but also the rest of their household. We used the German Socio-Economic Panel (SOEP) to calculate how much income the self-employed are able to sacrifice to achieve a subjective barely sufficient household income, which we interpret as the minimum level to maintain the standard of living. Our results suggest that full-time self-employed are typically the bread-earners in their households and that, as a consequence, even moderate income losses of the self-employed often lead to problems in maintaining the living standards of their households. Conditional on individual and household characteristics, the self-employed with employees are found to live in households that are less resilient to income losses. Furthermore, a negative correlation between falling short of the barely adequate household income and wellbeing was discovered. Self-employed in households with less than adequate incomes also reported higher concerns about social cohesion. These results have implications for policy - especially in light of the economic crisis induced by the pandemic.

## Introduction

In Germany, around four million individuals are self-employed. Due to the COVID-19 pandemic, many of these individuals are currently experiencing a slump in sales. Two in three self-employed individuals lost more than half of their sales - and around one in three self-employed individuals no longer generated any income at all (cf. Metzger, 2020). These numbers are basically corroborated by Block et al. (2020), who furthermore point to substantial revenue losses among the self-employed. Bertschek and Erdsiek (2020) report that for almost three in five solo self-employed (i.e., self-employed without any employees), monthly sales have plummeted by more than 75%. So far, most research about crises and income hardships of the self-employed refers to the self-employed themselves, which, however, allows only limited conclusions about the significance of income losses for their households. Since individual self-employment as well as the household environment are interwoven (see the excellent literature surveys provided by Bettinelli et al. (2014) and Carter et al. (2017)), it can be expected that the pandemic-induced crisis will negatively affect not only the self-employed themselves, but all members of their household.

Individuals and households smooth their consumption over time (Campbell & Deaton, 1989, Morduch, 1995). This might be especially practicable for paid employees, whose wages are usually rigid over time (cf. Goette et al., 2007). For the self-employed, in turn, consumption smoothing might be more challenging because incomes are more volatile and less downward rigid over time. Consider, for example, a pandemic-induced demand shock: Such a shock directly translates into lower incomes for the self-employed, while the paid employees are entitled their usual wages, short-time work compensation, or -in case of job loss- to unemployment benefits.^1^ Most of the social assistance rules are thus designed to dampen the effects of earnings shocks for paid employees, while the self-employed might even suffer a total loss of incomes in a very short time, which also affects their ability to smooth consumption and to maintain their standard of living over time.

The objective of this paper is to shed light on the relative importance of the incomes from self-employment in the household context. In addition, we analyzed how much income loss the self-employed could cope with to maintain the household’s standard of living. Finally, we address the implications for individual wellbeing of falling short of a barely adequate income level. For this purpose, we used German household data and examined the individual net income from self-employment, the net household income, as well as the subjective barely adequate household net income. Our empirical investigation suggests that the full-time self-employed are usually the bread-earners of households. However, we also observed that in the year 2018, 9% of full-time self-employed lived in households achieving an income below the barely adequate level and thus seem to struggle in maintaining the living standards, if incomes do not recover in the future. Half of all households will obtain a barely adequate income or less if the self-employed suffer income losses of about 37%. This strikingly reveals the importance of incomes obtained by the self-employed in the household context. Besides, we show that households with self-employed employers are less resilient to income cuts than the ones with solo self-employed. Moreover, our results point towards psychological as well as sociological consequences of falling short of the subjective barely adequate household income. The self-employed living in households with incomes below the barely adequate income level are most concerned about social cohesion and least satisfied with sleep or their lives in general. These results have implications for policy and pave ground for further research about the consequences of entrepreneurial crises in the household context.

## Data and methodology

### Data

We used the German Socio-Economic Panel - version 35 (SOEP, DOI: 10.5684/soep-core.v35). The SOEP is a longitudinal survey of more than ten thousand private households in Germany and is provided by the German Institute for Economic Research (DIW) Berlin. Basic data characteristics are described in Wagner, Frick and Schupp (2007) or Goebel et al. (2018). The SOEP contains information on demography, education, employment as well as the household.

In order to study the most recent available income information, we relied on the SOEP version 35, referring to year 2018. For the final analysis, we considered data restricted to full-time self-employed with an actual working time of at least 35 hours per week. The self-employed considered here are either freelancers or reported to be self-employed, whereas self-employed farmers remained unconsidered. We are particularly interested in the incomes of households, taking into account single person, single parent, and couple households with and without children.^2^ Furthermore, we only included self-employed individuals aged 18 to 65, while individuals in school were not unconsidered. In addition, we solely concentrated on self-employed with positive net incomes and positive household net incomes, who also reported a subjectively barely adequate monthly net income. Moreover, individuals with an individual labor net income exceeding the total household net income, i.e. due to indebtedness or child support in case of divorce or separation, were excluded from the analysis.

### Central variables

The most central variables in this study refer to information on net incomes. Specifically, we examined the current monthly labor net income of individuals as well as the net income of households.^3^ Besides, the SOEP questionnaire asks households about a barely adequate monthly net income, which we interpret as the minimum threshold level of income to maintain the current standard of living. The precise wording of the question is, ”The next question is about what you would consider a good or bad income in relation to your own personal circumstances and needs. What would you consider [...] a barely adequate income? (... EURO a month)”, asking households to indicate an appropriate net amount. This variable has the inherent advantage that it accounts for life models and living conditions and therefore reflects price differences across regions, variations in residential and living circumstances as well as heterogeneity in consumption.^4^ All income variables are measured in Euro.

We addressed the degree of resilience to income losses of the self-employed household member by calculating how much income the self-employed could sacrifice to maintain the household’s standard of living. In this regard, we examined the individual income of the self-employed, the current household income, and the subjective barely adequate income. We identified the following household types:

1. *Below margin households*: Households already achieving incomes below their barely adequate income level (household income < barely adequate household income).
2. *Marginal households*: Total income is exactly equal to the barely adequate income level (household income = barely adequate household income). These households cannot sustain income losses.
3. *Above margin households*: Households with total incomes above the barely adequate level (household income > barely adequate household income), which are not able to sacrifice all of the income from self-employment (household income - barely adequate household income < individual labor income of the self-employed). For this group of households, we calculate the maximum bearable income loss from self-employment so that the household obtains exactly the barely adequate income level ($\frac{\,{{\mbox{household income}}}-{{\mbox{barely adequate household income}}}}{{{\mbox{individual labor income of the self-employed}}}\,}\times 100$).
4. *Resilient households*: This group of households is able to sacrifice the income from self-employment in general (household income - individual labor income of the self-employed ≥ barely adequate household income). This is the case, for example, if the partner covers the household’s subjective adequate income entirely with wages or salaries from paid employment. These households are considered particularly resistant to income shocks to the self-employed.

For instance, consider a household with a barely adequate household income of 3,000 Euros. If the household has a self-employed bread-earner with income of 3,500 Euros and no additional income, then this above margin household could sacrifice 500 Euros or 14.3% of income from self-employment to achieve exactly the barely adequate income level. Any further income losses imply that the household will not be able to retain its standard of living in the long-run. Alternatively, consider that the partner adds 500 Euros to total household income.^5^ Total household income then sums up to 4,000 Euros. In this case, the household is able to bear an income cut of the self-employed person by 1,000 Euros or 28.6%. Finally, if the partner earns 3,500 Euros or more in paid employment, the household is able to forfeit all income from self-employment and is thus defined as a resilient household.

## Results

### The relevance of income from self-employment within households

All results presented in this section are weighted with the individual weighting factor provided by the SOEP. Calculations are based on 281 solo self-employed and 290 employers, which are representative of 826,634 self-employed without and 738,745 self-employed with employees. It is crucial to distinguish between these two types of self-employment because the incomes of the two groups differ significantly. In our sample, the median full-time solo self-employed obtained a net income of 1,800 Euros, while the median employer achieved a net labor income of 3,000 Euros. Furthermore, the average individual net income of the solo self-employed (2,313 Euros) was smaller than the one of the self-employed with employees (3,535 Euros). These results are in line with the stylized facts about the income situation for these different types of self-employment (cf. Maier & Ivanov, 2018, Schneck, 2020, Sorgner et al., 2017).

**Fig. 1:**
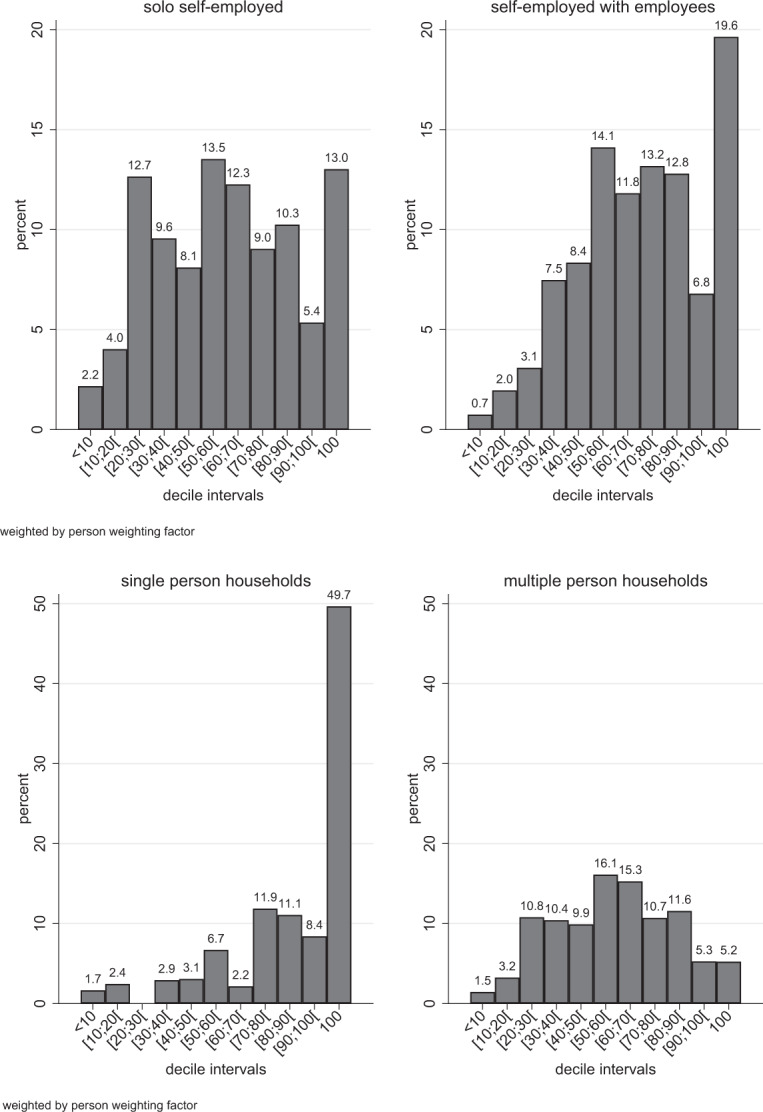
Share of individual net income in net household income in percent

Focusing on the importance of the net incomes of the self-employed in the household context, we found that the self-employed with employees tended to not only generate higher incomes, but they also contributed more money to their total household income than the ones without employees (cf. upper panel of Fig. 1). For example, a fifth of all self-employed with employees (19.6%) generated total household income, while less than one in seven (13.0%) of solo-self-employed managed to do so. Furthermore, about each third solo self-employed individual (36.5%) was not able to provide half or more of total household income. Among the self-employed with employees, the corresponding share was considerably smaller (21.6%). On average, the self-employed contributed about two thirds to total household net income (63.8%).

We distinguished between single and multi-person households because further household members might obtain additional labor incomes or pensions. In addition, relative to single person households, multi-person households are entitled to different social support payments, such as child allowance, or are subject to differences in tax regulations, which affect the calculation of net incomes. Figure 1 (lower panel) shows that the self-employed usually generated the majority of total income in single person households, which implies that labor income is the primary source of household income. In nine out of ten single person households, the self-employed contribute more than half of the total household income. The corresponding median net labor income of the self-employed amounted to 94.8% of total household income. In multiple-person households, two in three self-employed contributed at least half of the total household income. The corresponding median share of the net income contributed by the self-employed to the total household net income amounted to 57.9%. The relative importance of the incomes from self-employment with respect to the net household income was thus substantial. In fact, the presented results suggest that full-time self-employed are usually the main bread-earners in their households.

### Income loss and living standards

**Fig. 2:**
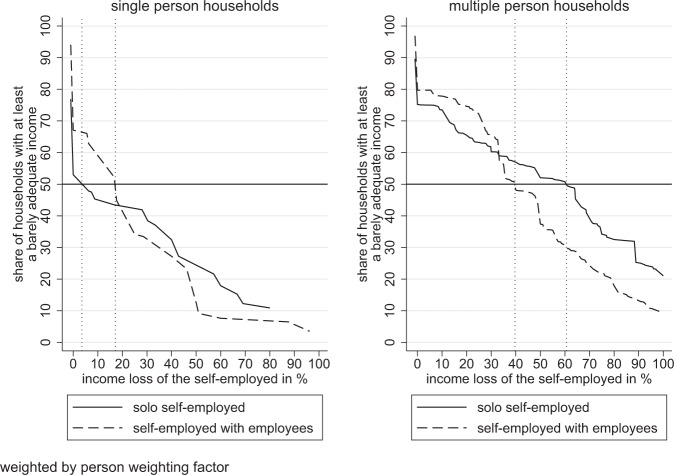
Share of households with at least a barely adequate household income if the self-employed experience income cuts

To examine how high is the net income cut that is still bearable, we compared the household income with the barely adequate household income surveyed in the SOEP.^6^ Specifically, we referred to the four household types described in section 2.3. Even before the start of the pandemic, in year 2018, 23.1% of the solo self-employed in single-households reported that their current household income falls short of a barely adequate income (below margin household). In contrast, the share of self-employed with employees in below margin single households was considerably lower (5.9%). Since this group of households already did not obtain the barely adequate income level, the lines in Fig. 2 (left panel) start at 76.9% (solo self-employed) and 94.1% (self-employed with employees), respectively. The likelihood of living in a below margin household was considerably lower for the solo self-employed if the household consisted of more individuals (10.3%, cf. right panel of Fig. 2). We thus observe substantial differences by household size. This can be explained by other household members contributing to the household income.

The sharp drop of the lines at hypothesized income cuts of 0 in Fig. 2 indicates that many households could not sustain any income cuts because household income was equal to the reported barely adequate level. In fact, 17.2% of all considered households with self-employed were living in marginal households and obtained exactly the income to maintain their standard of living. The steeper decline at hypothesized income cuts of 0 in the left panel implies that the share of marginal households was larger in single when compared to multiple member households. The graph also displays that the share of households achieving the barely adequate income level decreased with increasing hypothesized income cuts of the self-employed (above margin households). In this regard, Fig. 2 shows that single person households are more vulnerable to income losses than multiple person households. Including the below margin as well as the marginal households, less than half of the solo self-employed in single person households could not cope with modest income cuts of 5% and still maintain their households’ living standards. Half of all self-employed with employees living alone started to struggle in maintaining their living standards in case of hypothesized income cuts of less than 20%. Even moderate income losses thus translated into problems in maintaining the living standards. In households with more members, the particular income loss threshold values were much larger. To be precise, half of multiple member households with solo self-employed could cope with income losses of about 60%, while the ones with self-employed employers could bear income cuts of about 40%. When considering the entire sample, half of all households could cope with income cuts up to 37% of the total labor income of the self-employed.

Figure 2 also reveals an intersection point of the cumulative curves. Relative to employers, a higher share of solo self-employed individuals was not able to forego moderate incomes to maintain their living standards. The cumulative distribution then intersects and from this point onward, the cumulative share of solo self-employed dominates the one of the employers. This suggests that households with self-employed employers would suffer relatively more from severe income losses and tend to be more likely to struggle in maintaining their living standards. Based on our calculations, 14.6% of self-employed individuals lived in households that could sustain a complete loss of income from self-employment. The share of resilient households was larger in multiple than in single person households.

### Factors describing the resilience of households

Are self-employed with certain characteristics more likely to maintain their living standards? To examine this question, we created an ordinal variable reflecting the four household types mentioned in section 2.3, which indicates the degree of resilience of the household to income losses. The variable is ordered from not resilient at all to very resilient to income losses of the self-employed. In analogy, we also created an additional ordinal variable to gather more information about the resilience of households achieving incomes above the barely adequate level. Precisely, we created categories for above margin households describing the maximum bearable income loss in increments of 10%, where higher values indicate that the household could sustain higher income cuts from self-employment. Then, we estimated an ordered probit model to examine the relationship between characteristics of the self-employed as well as his/her household and resilience. The self-employed in the considered sample were usually solo self-employed (52.1%), male (71.0%), living in couple households (31.8% without children and 38.1% with children), had an intermediate/higher secondary (46.0%) or tertiary (38.8%) educational background, were, on average, 48.6 years old, and lead the businesses, on average, for 13.1 years. For descriptive statistics, see Table A.1 in the appendix. With respect to occupation, every fifth self-employed individual worked in the health, social services, teaching and education sectors, followed by self-employed with occupations related to raw material extraction, production and manufacturing as well as business organization, accounting, law and administration (around 16%).^7^

The negative coefficient of self-employed with employees in specification (1) of Table 1 indicates that households with self-employed employers were, ceteris paribus, less resilient to income cuts than the ones with solo self-employed. With respect to household size, the estimation results show that single person households were the least resilient to income losses of the self-employed. Also single parent households were less resilient than couple households with and without children. Additional incomes of partners thus helped to make couple households less sensitive to income shocks of the self-employed.^8^ Higher education and labor market experience were positively correlated with resilience. Moreover, older self-employed were more likely to struggle in maintaining their living standards when compared to younger ones. In addition, the positive coefficient indicates that households with male self-employed were more resilient to income losses than the ones with female self-employed. The results presented in Table 1 are qualitatively robust to the inclusion of dummy variables describing occupational activities of the self-employed. This implies that controlling for low/high income occupational tasks does not change the main results.

**Table 1:** Ordered probit estimates describing the resilience to income losses

	(1)	(2)
	Resilience to income losses	
	4 categories^a^	13 categories^b^
Solo self-employed	Reference category	
Self-employed with employees	−0.085***	−0.159***
	(0.002)	(0.002)
Household size		
Single person household	Reference category	
Couple without children	0.582***	0.769***
	(0.003)	(0.002)
Single parent	0.249***	0.231***
	(0.005)	(0.004)
Couple with children	0.604***	0.678***
	(0.002)	(0.002)
Education		
Primary/lower secondary education	Reference category	
Intermediate/higher secondary education	0.315***	0.157***
	(0.003)	(0.003)
Tertiary education	0.607***	0.476***
	(0.003)	(0.003)
Labor market experience in years		
Experience in full-time work	0.018***	0.004***
	(0.000)	(0.000)
Experience in part-time work	0.034***	0.018***
	(0.000)	(0.000)
Unemployment experience	−0.087***	−0.092***
	(0.000)	(0.000)
Tenure (leading the same business)	0.018***	0.013***
	(0.000)	(0.000)
Demographics		
Age	−0.020***	−0.005***
	(0.000)	(0.000)
Male	0.082***	0.110***
	(0.002)	(0.002)
cut1	−0.984***	−0.694***
	(0.007)	(0.007)
cut2	−0.155***	0.124***
	(0.007)	(0.007)
cut3	1.616***	0.214***
	(0.007)	(0.007)
cut4		0.369***
		(0.007)
cut5		0.569***
		(0.007)
cut6		0.853***
		(0.007)
cut7		1.090***
		(0.007)
cut8		1.203***
		(0.007)
cut9		1.431***
		(0.007)
cut10		1.633***
		(0.007)
cut11		1.826***
		(0.007)
cut12		1.923***
		(0.007)
Number of weighted observations	1,485,505	
Log-likelihood	−1,562,189.8	−3,443,077.0
Pseudo *R*^2^	0.068	0.038

Heteroskedasticity-robust standard errors in parentheses

+*p* < .10; *p < .05, **p < .01, ***p < .001

^a^Specification (1): Dependent variables consists of 4 categories. In angular brackets: shares with respect to the considered sample.

1 Below margin household 〈9.25%〉

2 Marginal household 〈18.20%〉

3 Above margin household 〈57.86%〉

4 Resilient household 〈14.68%〉

^b^Specification (2): Dependent variables consists of 13 categories. In angular brackets: shares with respect to the considered sample.

1 Below margin household 〈9.25%〉

2 Marginal household 〈18.20%〉

3-12 Above margin household:

3 Bearable income cut $\left.{{{{{\rm{in}}}}}}\ \right]$0;10%] 〈2.81%〉

4 Bearable income cut $\left.{{{{{\rm{in}}}}}}\ \right]$10;20%] 〈5.04%〉

5 Bearable income cut $\left.{{{{{\rm{in}}}}}}\ \right]$20;30%] 〈7.02%〉

6 Bearable income cut $\left.{{{{{\rm{in}}}}}}\ \right]$30;40%] 〈10.40%〉

7 Bearable income cut $\left.{{{{{\rm{in}}}}}}\ \right]$40;50%] 〈8.50%〉

8 Bearable income cut $\left.{{{{{\rm{in}}}}}}\ \right]$50;60%] 〈3.89%〉

9 Bearable income cut $\left.{{{{{\rm{in}}}}}}\ \right]$60;70%] 〈7.33%〉

10 Bearable income cut $\left.{{{{{\rm{in}}}}}}\ \right]$70;80%] 〈5.77%〉

11 Bearable income cut $\left.{{{{{\rm{in}}}}}}\ \right]$80;90%] 〈4.86%〉

12 Bearable income cut $\left.{{{{{\rm{in}}}}}}\ \right]$90;100%$[ \langle 2.22 \% \rangle$

13 Resilient household 〈14.68%〉

### Household income situation and individual wellbeing of the self-employed

**Fig. 3:**
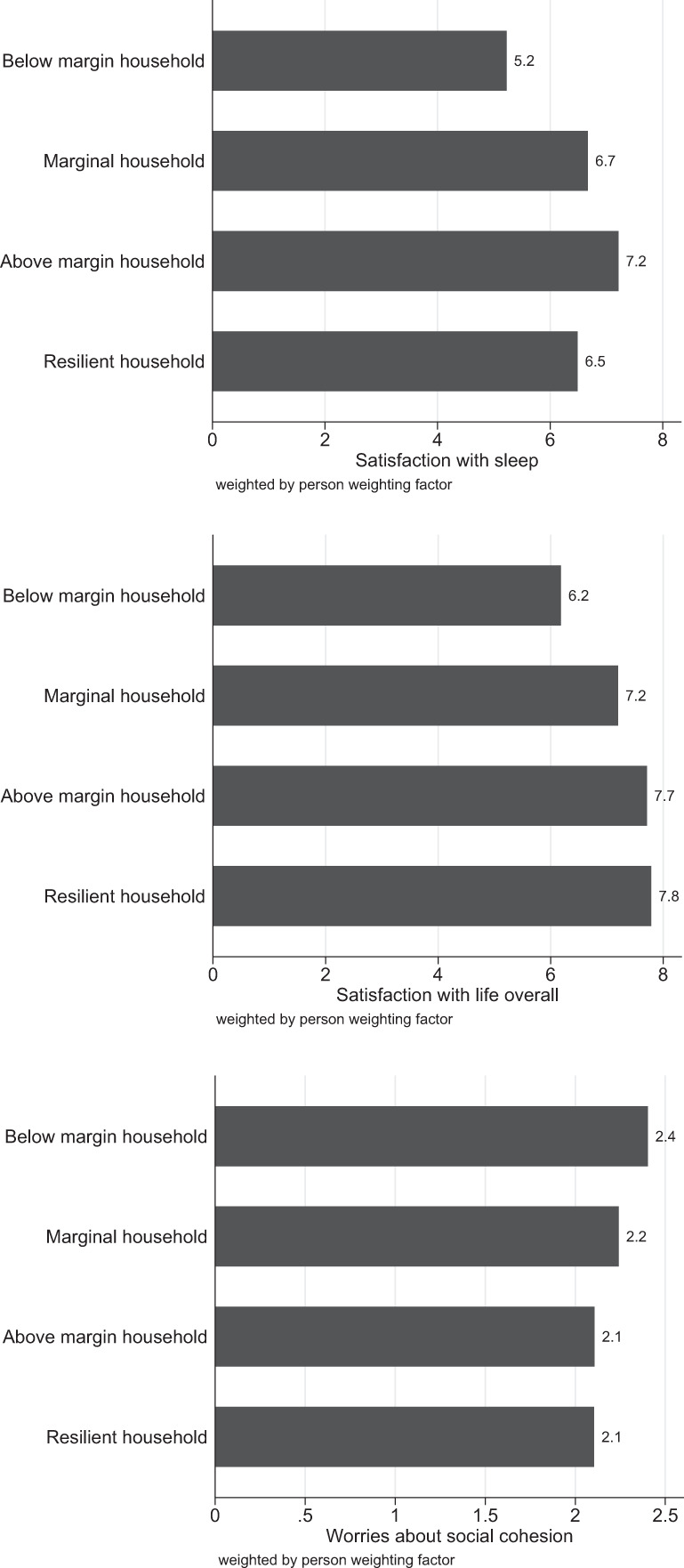
Average reported satisfaction and worries about social cohesion of the self-employed by income situation of the household

Our results show that the full-time self-employed were usually the bread-earners within households. For this reason, income cuts frequently lead to incomes below a subjectively barely adequate income level. In case of income cuts, individuals might maintain their living standards in the short-run, but if the incomes do not recover or if the other household members do not find a higher paid job while expenses remain stable, the living standards cannot be maintained in the mid- to long-run without (over)indebtedness. The fear of a decline in living standards might cause stress and might have adverse psychological consequences. Based on a descriptive analysis of the underlying sample, the self-employed with observed household incomes below the subjectively barely adequate level reported lower satisfaction with sleep and exhibited lower levels of overall life satisfaction (Fig. 3).^9^

In the mid- to long-run, individuals might realize a decline in the standards of living if incomes do not recover. In such cases, further adverse effects at the individual and household level can be expected. For example, one might think about marital crises or separations. Besides, it might be speculated about spillover effects, such as dissatisfaction with policy, the economic system, or society as a whole. Descriptive analysis revealed that more than half of all self-employed individuals who did not obtain a barely adequate household income reported that they are very concerned about social cohesion.^10^ Among those with household income levels above the barely adequate level, 24.8% of the self-employed stated that they were very concerned about social cohesion. Struggling in maintaining the standard of living thus seems to cause spillover effects into various dimensions of life and individual perceptions. Figure 3 corroborates that worries about social cohesion were, on average, highest among self-employed living in below margin households.

We estimated ordered probit regressions to assess potential negative consequences after accounting for individual as well as household characteristics (cf. Table 2). Even after controlling for household size, education, labor market experience, and demographics, the self-employed living in below margin households were significantly less satisfied with sleep than self-employed in households achieving at least the barely adequate income level. Analogously, the same holds for satisfaction with life overall. The most resilient self-employed living in households that were able to forgo all self-employment income tended not to be the most satisfied. With respect to worries about social cohesion, we estimated significantly negative coefficients when incomes were at least equal to the current cost of living, which implies that the self-employed in below margin households are the most concerned about social cohesion. The results presented in Table 2 are qualitatively robust to the inclusion of the resilience indicator with 13 categories.

**Table 2:** Ordered probit estimates describing the consequences of falling short of the barely adequate income level

	(1)	(2)	(3)
	Satisfaction with		Worries about
	Sleep^*a*^	Life overall^*a*^	Social cohesion^*b*^
Household type			
Below margin household	Reference category		
Marginal household	0.468***	0.142***	−0.259***
	(0.004)	(0.004)	(0.004)
Above margin household	0.698***	0.418***	−0.508***
	(0.004)	(0.004)	(0.004)
Resilient household	0.340***	0.319***	−0.425***
	(0.004)	(0.004)	(0.004)
Solo self-employed	Reference category		
Self-employed with employees	0.067***	−0.050***	0.004*
	(0.002)	(0.002)	(0.002)
Household size			
Single person household	Reference category		
Couple without children	0.131***	0.393***	0.106***
	(0.002)	(0.002)	(0.003)
Single parent	0.435***	0.236***	0.513***
	(0.004)	(0.004)	(0.005)
Couple with children	0.285***	0.494***	0.122***
	(0.002)	(0.002)	(0.003)
Education			
Primary/lower secondary education	Reference category		
Intermediate/higher secondary education	0.024***	0.506***	−0.665***
	(0.003)	(0.003)	(0.003)
Tertiary education	0.031***	0.682***	−0.560***
	(0.003)	(0.003)	(0.003)
Labor market experience in years			
Experience in full-time work	0.011***	0.025***	−0.002***
	(0.000)	(0.000)	(0.000)
Experience in part-time work	−0.019***	−0.020***	−0.006***
	(0.000)	(0.000)	(0.000)
Unemployment experience	−0.080***	−0.065***	0.033***
	(0.000)	(0.000)	(0.000)
Tenure (leading the same business)	−0.018***	−0.007***	0.017***
	(0.000)	(0.000)	(0.000)
Demographics			
Age	0.006***	−0.012***	−0.003***
	(0.000)	(0.000)	(0.000)
Male	−0.225***	−0.313***	0.000
	(0.002)	(0.002)	(0.002)
cut1	−2.495***	−2.750***	−1.987***
	(0.011)	(0.011)	(0.008)
cut2	−0.962***	−2.058***	−0.141***
	(0.007)	(0.008)	(0.008)
cut3	−0.555***	−1.468***	
	(0.007)	(0.008)	
cut4	−0.247***	−1.134***	
	(0.007)	(0.008)	
cut5	0.201***	−1.038***	
	(0.007)	(0.008)	
cut6	0.427***	−0.640***	
	(0.007)	(0.008)	
cut7	0.940***	−0.274***	
	(0.007)	(0.008)	
cut8	1.566***	0.315***	
	(0.007)	(0.008)	
cut9	2.200***	1.334***	
	(0.007)	(0.008)	
cut10		2.477***	
		(0.008)	
Number of weighted observations	1,485,505	1,485,505	1,484,644
Log-likelihood	−2,971,210.9	−2,491,222.8	−1,303,437.3
Pseudo *R*^2^	0.038	0.055	0.044

Heteroskedasticity-robust standard errors in parentheses

+*p* < .10; *p < .05; **p < .01; ***p < .001

^a^Variable ranges from 0 (completely unsatisfied) to 10 (completely satisfied)

^b^Variable with three outcomes: 1: no worries, 2: some worries, 3: very concerned

## Discussion

From an economic perspective, entrepreneurs and the self-employed create jobs and contribute to economic prosperity. Our paper contributes the finding that self-employed are the bread-earners in their households and thus usually secure the standards of living not only for themselves but also for the entire households. In other words, the self-employed not only create stable jobs but also provide stable family relationships, which illustrates the economic and social importance of the self-employed in society. However, the self-employed experienced severe slumps in sales during the pandemic. Based on a flash survey conducted in March/April 2020, Metzger (2020) shows that two-thirds of all self-employed respondents suffered sales cuts of at least 50%. Our results suggest that roughly six in ten self-employed would not be able to maintain their standard of living in the long-run if the sales losses directly translate into income cuts by one half. Single person households would be much more severely affected when compared to households with more than one members. Moreover, Metzger (2020) shows that one in three of surveyed self-employed individuals no longer generated any sales during the first months of the pandemic. The results presented here reveal that 14.6% of considered self-employed individuals live in households that could maintain their standards of living despite complete loss of income from self-employment. The share of resilient households was larger in multiple when compared to in single person households. Obviously, other sources of income helped to maintain the standards of living in times of crisis for the self-employed.

During the pandemic, policymakers faced a trade-off between economic and social disease-related interventions. The imposed policy measures in Germany included, among others, distancing rules and the closure of companies with close customer contact. Some self-employed had purchased insurance for business closure due to the risk of epidemics and/or infections, but the legal situation regarding the liability of business closure or business interruption insurance policies (*Betriebsschließungs-/Betriebsausfallversicherungen*) in the pandemic has not yet been conclusively clarified. Few entrepreneurs actually have been voluntarily insured against unemployment (Jahn & Oberfichtner, 2020) and therefore might receive unemployment benefits. Others were completely surprised by the situation and were socially as well as economically unprotected. The pandemic and the measures associated with it, thus, directly led to lower incomes for many self-employed. In contrast, paid employees are entitled their usual wages, short-time work compensation^11^, or -in case of job loss- to unemployment benefits. In other words, social security schemes are broadly designed for employees, not the self-employed. However, the German Minister of Economics Affairs announced at a press conference in March 2020 that ”[n]o healthy company should go bankrupt due to corona”.^12^ Therefore, German policy introduced emergency aid programs for the self-employed, usually in form of one-off lump sum grants, such as the so-called *Soforthilfe, November- und Dezemberhilfe*(also see Block et al., 2020). In addition, tax relief measures were adopted and self-employed individuals were given easier access to basic unemployment benefits (the so-called *Arbeitslosengeld II*) without having to undergo a wealth check. However, basic unemployment benefits might not help to secure the living standards in entrepreneurial households, but they can secure the absolute subsistence level. In sum, governments introduced a magnitude of measures to secure at least an absolute basic level of income for the self-employed and their households. Whether the aid programs have helped to ensure business survival and to maintain the living standards of entrepreneurial households can only be examined with more current data and remains a promising avenue for future research.

The results presented above show that the self-employed with employees are less resilient to income cuts than the solo self-employed. This interesting finding might have to do with certain entrepreneurial attitudes, with solo self-employed having few obligations to others if they need to cut costs. The self-employed with employees, in turn, might feel responsible for their employees and might even forego their own income in order to save jobs (see, e.g., Kraus et al., 2020). However, labor hoarding makes sense in temporary crises because the company can recover faster after the downturn with the already trained and qualified employees, which then increases the (household) income of the self-employed.^13^ If the crisis is too severe, the self-employed may also decide to lay off workers if they are struggling in maintaining their standard of living, which then causes adverse effects for the employees and their families. During the pandemic, it might also be possible that both, entrepreneurs (due to sales losses) and employees (due to short-time work or lay-offs) are unable to maintain their standard of living during the pandemic. We encourage further research on these phenomena.

Another rationale for the result that self-employed with employees are less resistant to income losses is that these self-employed achieve higher absolute incomes (see Maier & Ivanov, 2018, Sorgner et al., 2017, and the results presented above) and usually contribute more to household income than the solo self-employed (see Fig. 1). As a result, other sources of household income tend to be lower and therefore cannot compensate for significant income reductions of the self-employed. In turn, incomes are more evenly distributed in households with solo self-employed than in households with self-employed with employees. The lower contribution of solo self-employed to household incomes or the higher degree of income diversification, respectively, increases resilience in times of crisis. However, the pandemic is a very unique crisis, which is also expected to have negative effects on other sources of income within households. For this reason, we need follow-up studies with more recent data that will allow us to examine overall income losses and living standards within households during the pandemic. One way forward is the analysis of income and labor-related risks of partners or spouses (Peluffo & Viollaz, 2021). Some branches have been hit more heavily by the pandemic itself or by policy-induced measures to contain the pandemic than others. For this reason, special attention should be paid to the fact that income and labor-related risks also depend on whether partners work in identical or different industries (cf. Peluffo & Viollaz, 2021).

The results presented in this paper show that an income below the barely adequate level entails stress (lower satisfaction with sleep) and lower life satisfaction among the self-employed. Concerns about social cohesion are also registered significantly more often by self-employed with an income below the subjectively just sufficient level. This implies that troubles in securing the standard of living cause spillover effects into various domains of life. In this regard, one might speculate about behavioral or election-related responses as well. In the aftermath of the pandemic, data might be available to address the relationship between living standards, satisfaction, and potential spillover effects with income loss in self-employed households in more detail. In this regard, it is also interesting to distinguish between the self-employed, who already had problems securing their standard of living before the crisis, and those for whom income problems arose during the crisis.

Although this study provides insights on whether households with self-employed are able to maintain their living standards in the very short-run, this paper has not yet addressed the duration of income cuts, labor market responses of household members such as starting a (side-)job as a paid employee, or possible adjustments in consumption. Another promising field of research is the consideration of assets and property, which have considerable effects on the living standards in the long-run and the possibilities to weather a crisis. Moreover, differences in consumption levels or heterogeneity in living standards are not yet considered. Future studies might address these issues in greater depth.

## Conclusion

With German household data, we show that full-time self-employed individuals are usually the bread-earners in their households. Economic shocks, which directly translate into the economic situation of the self-employed, thus, have considerable effects at the household level. Our study contributes an analysis on the resilience of entrepreneurial households by examining how much income from self-employment could be sacrificed to achieve a subjective barely adequate level of household income, which is sufficient to maintain the current living standards. The calculations suggest that half of all households can cope with income cuts of up to 37% of the total labor income of the self-employed. Based on the results presented here and the significant slumps in sales (cf. Bertschek & Erdsiek, 2020, Block et al., 2020, Metzger, 2020) as well as income (Graeber et al., 2021), one might speculate that many households with self-employed struggle in maintaining their standards of living during the pandemic. Every seventh of the considered full-time self-employed lived in a resilient household that can sustain a complete loss of income from self-employment. Moreover, we found that income losses represent a higher burden for the self-employed with employees when compared to the solo self-employed. Finally, our results point towards negative effects of falling short of the barely adequate income level: Individual stress (measured as satisfaction with sleep) and satisfaction with life were significantly lower among the self-employed in households falling short of the barely adequate income level. Also, spillover effects on society can be expected because households with incomes below the income level to maintain their standards of living report to be more concerned about social cohesion.

This paper can be understood as a call for more research on the consequences of income hardships among the self-employed in the context of households. Specifically, we encourage studies, which examine the resilience and behavioral responses of self-employed in times of crises at the individual (see, e.g., Fallon & Lucas, 2002), but also at the household level. In this line, it is of importance whether and how household incomes recover after shocks. Moreover, one could speculate that some self-employed are reassessing their entrepreneurial decision and place more emphasis on steady, more rigid income streams from paid employment and consequently shut down their businesses. We therefore suggest that studies on business closures should not only focus on (the usual) business characteristics but also try to consider the income situation of the entrepreneurial household. Moreover, the lack of financial and social security for the self-employed, which became apparent during the pandemic, can affect entrepreneurial intentions in general, which might hamper start-up activities as well as the search for potential successors - both within and outside the family. Addressing these issues is not only interesting for the fields of entrepreneurship and family business research, but will also help to understand recovery processes, employment growth, and market concentration in the aftermath of the pandemic. Furthermore, the analysis of possible spillover effects of income hardship in entrepreneurial households represents a promising avenue for further research and will provide valuable insights for policymakers and society alike. Although dramatic for individuals, households as well as the economy as a whole, the recent pandemic-induced crisis offers a natural experiment, which might help researchers to gather data and address the effects of income cuts for individuals, households, the economy, and society.

## Appendix

Table A.1

**Table A.1:** Descriptive statistics when considering personal weighting factor

Variable	Mean	Standard deviation
Solo self-employed	0.521	0.500
Self-employed with employees	0.479	0.500
Household		
Single person household	0.252	0.435
Couple without children	0.318	0.466
Single parent	0.048	0.215
Couple with children	0.381	0.486
Education		
Primary/lower secondary education	0.152	0.359
Intermediate/higher secondary education	0.460	0.499
Tertiary education	0.388	0.488
Labor market experience in years		
Experience in full-time work	22.224	10.690
Experience in part-time work	2.070	4.001
Unemployment experience	0.814	2.780
Tenure (leading the same business)	13.098	9.091
Demographics		
Age	48.584	9.522
Male	0.710	0.454
Number of weighted observations	1,485,505	
Number of observations	552	

## Footnotes

1 Entrepreneurs in Germany can insure themselves voluntarily against unemployment, but only few actually do so. Between 2013 and 2018, the number of voluntarily insured self-employed decreased from 145,000 to 76,000 (Jahn & Oberfichtner, 2020).

2 Note that we abstained from considering so-called ”other household combinations” and ”more generation households”. More generation households describe a form of intergenerational cohabitation of (non-)related persons living together. In some cases, this form of cohabitation implies that all household members secure their individual and joint livelihood. However, multigenerational households could also be a form of cohabitation between different generations in which a pensioner is financially independent of the other household members. Since we were not able to precisely determine the degree of financial interdependence of household members, we omitted these households from our analysis. This degree of uncertainty about financial dependence also applied to ”other household combinations”. While couples might be assumed to earn their living jointly, members in these so-called other household combinations may secure their livelihood individually or jointly.

3 Respondents are asked to include regular payments such as pensions, housing allowance, child benefit, or alimony to total net income.

4 The measure also has some disadvantages. For example, it is well known that the individual perception of *adequate* is a function of relative standing within a particular reference group. It includes inherent subjective needs, wants as well as pleasures and is not an objective measure such as the poverty line or other absolute measures applied in poverty research.

5 Note that the survey refers to the monthly net household income of all household members, which is not limited to labor income, but comprises any source of (regular) income.

6 Note that we assumed that all other sources of household income remained constant. In the current pandemic, such an assumption can certainly be viewed critically. If other household incomes decrease as well, even smaller income cuts might lead to household incomes below the barely adequate level and to a loss of the living standards.

7 Information is based on the German classification of occupations 2010 (*KldB10*), which is closely related to the International Standard Classification of Occupations 2008 (*ISCO-08*). Due to missing occupational classification values, the number of observations decreased to 526 observations or 1,397,483 weighted observations, respectively.

8 Note, however, that the ceteris paribus consideration does not take into account that other household members might suffer income cuts as well during severe crises.

9 Satisfaction levels are measured on an 11-items Likert-scale ranging from 0 (completely unsatisfied) to 10 (completely satisfied). The reported descriptive statistics are based on weighted observations.

10 We examined the weighted number of observations. Worries are measured on a three items scale, including the statements ”no worries”, ”some worries”, and ”very concerned”.

11 Before the pandemic, the Federal Employment Agency paid 60% of lost wages in the case of short-time work (67% for parents). During the pandemic, short-time allowance was increased. From the fourth month of eligibility, short-time allowance for childless employees was increased to 70% and from the seventh month of receipt to 80% of lost wages. For employees with children, the increase was more generous, amounting to 77% of lost wages from the fourth month and 87% from the seventh month.

12 See the joint press release of the Federal Ministry for Economic Affairs and Energy and the Federal Ministry of Finance on March 13, 2020: https://www.bmwi.de/Redaktion/EN/Pressemitteilungen/2020/20200313-protective-shield-for-employees-and-companies.html, accessed March 26, 2021.

13 In this regard, Germany’s short-time work scheme helps to keep employment levels stable and to retain valuable employees during downturns (cf. Cahuc, 2019). For employers, the social security contributions have been waived during the pandemic. See International Monetary Fund (2020) for a brief but comprehensive overview about the German short-time work scheme, the main legal changes during the pandemic, and some statistics showing that short-time work has been used extensively across various sectors during the pandemic.
